# Posture control in Pusher syndrome: influence of lateral semicircular canals

**DOI:** 10.1016/S1808-8694(15)31197-6

**Published:** 2015-10-20

**Authors:** Taiza Elaine Grespan dos Santos Pontelli, Octavio Marques Pontes-Neto, José Fernando Colafêmina, Draulio Barros de Araújo, Antonio Carlos Santos, João Pereira Leite

**Affiliations:** 1 Specialist in Neurological Physical Therapy, physical therapist, post-graduate studies under course.; 2 Physician, Residence Program in Neurology, Residence program in Neurophysiology, Post-graduate studies in Neurology under course.; 3 Assistant Professor, Ph.D.; 4 Bachelor in Physics, Ph.D. studies under course in Medical Physics.; 5 Assistant Professor, Ph.D.; 6 Post-graduate studies in Neurology.

**Keywords:** Pusher syndrome, vestibular system, postural control

## Abstract

Pusher syndrome is an interesting disorder of balance in patients with encephalic lesions characterized by the peculiar behavior of actively pushing away from the non-hemiparetic side and resisting against passive correction, with a tendency to fall toward the paralyzed side. The role of vestibular system on the pushing behavior is not clear. **Aim**: To evaluate horizontal semicircular canal function in patients with Pusher syndrome, using caloric and rotation tests. **Study Design**: Observational prospective. **Material and Method**: We evaluated 9 inpatients with stroke and Pusher syndrome at the neurological unit of HCFMRP-USP. We applied neurological and neuropsychological exams, NIHSS, Scale for contraversive pushing (SCP), caloric and rotation tests. **Results**: We evaluated 9 patients (5 men) with mean age of 71.8 ± 5.9 and mean NIHSS of 18.33. Three patients presented contralateral directional preponderance on caloric test and we found four patients with directional preponderance on analysis of the slow phase velocity of rotation test response. **Conclusion**: Our findings indicate that a dysfunction of semicircular canals does not seem to be relevant for the clinical manifestations of the Pusher syndrome.

## INTRODUCTION

The understanding of vertical posture control mechanisms is extremely important both for physiological situations and in view of specific neurological syndromes. The perception of vertical position has its only absolute reference - the acceleration of gravity ^1^. Its conception would depend on recognition of body position in relation to objects and through vision, gravitational perceptive and proprioceptive afference. Normal people have the skills to visually position themselves in vertical line almost without angulations in relation to the Earth, assessed through visual vertical subjectivity (SVV)^2^. They are also capable of having their bodies oriented with the vertical line correspondent to the use of sight, assessed by means of the postural vertical subjectivity (SVP)^3^. These components are indispensable for the static and dynamic posture control.

Human static posture is maintained through a program of central posture assisted by many sensorial modalities, especially those of vestibular, visual, muscular, cutaneous and interoceptive origin 4, 5, 6. Together, these systems interact for the stabilization and posture representation of the body.

Many different balance and body verticalization affections can be attributed to paresis resulting from encephalic damage 7, 8. In such cases, the patients normally use the non-paretic limb to correct his/her posture 7, 8. Other balance affections are present in Wallemberg syndrome, thalamic asthasia and Pusher syndrome.

Pusher syndrome has been considered one of the most intriguing affections of posture control found in patients with encephalic damage. Patients with this syndrome, rather than pulling themselves in order to sustain their bodies, tend to push towards the paretic side and use the non-affected limb. If static, both seated and in orthostatic position, they present a contralateral inclination of the encephalic lesion. In view of the passive correction, these patients use the non-affected side to resist correction, reporting lack of confidence and fear of fall ^9^.

This syndrome was initially described by Davies, in 1985, who suggested the association of cerebral vascular accidents (CVA) on the right hemisphere and neuropsychological symptoms of heminegligence and anosognosia as part of this syndrome ^9^. Later studies in patients with Pusher syndrome allowed the dissociation of posture control of neuropsychological symptoms and found frequent left side damage 10, 11, 12.

Overlapping imaging studies by magnetic resonance (MRI) or computed tomography (CT scan) indicate the posterior ventral nucleus and the lateral-posterior nucleus of the posterior-lateral thalamus as critical structures to manifest the pushing behavior ^11^. Other possible and already described areas are: internal capsule, supplementary motor area, upper parietal lobe, pallid globe, and parietal-insular vestibular cortex 13, 14, 15.

Up to recently, Pusher syndrome had only been described in patients with CVA who presented symptom resolution within approximately six months. However, in a study carried out in our neurological emergency unit we identified the association of Pusher syndrome and other etiologies (cranial trauma and cerebral metastases), with times of resolution that were apparently briefer ^12^.

Patients with Pusher syndrome present a significant body orientation disorder in relation to gravity acceleration ^16^. When seated down on a reclining chain on the frontal plan, with lateral protection and without visual support, these patients sit vertically when positioned with inclination ipsilateral to the damage ^18^. However, the perception of visual verticality is not affected, because they are capable of correctly positioning a light beam in the vertical position.

The role of the vestibular syndrome in the posture affection of Pusher syndrome is still not properly clarified. The influence of semicircular canals in the position and angular acceleration of the cephalic segment in relation to the body suggests that this system may eventually contribute to vertical orientation. Alternatively, the imbalance of Pusher syndrome could be resultant from encephalic dysfunctions, without participation of the vestibular system. In this study, we aimed at assessing the role of horizontal semicircular canals in the clinical expression of Pusher syndrome, through the application of caloric and rotation tests.

## MATERIAL AND METHOD

We assessed 9 patients with diagnosis of CVA that presented Pusher syndrome and were hospitalized in the Neurology Emergency unit, Hospital das Clínicas, Medical School, Ribeirão Preto, University of Sao Paulo. All patients were submitted to clinical neurological assessment and evaluation of neuropsychological symptoms of heminegligence and anosognosia. Patients were classified as presenting spatial heminegligence when they presented clear evidence of spontaneous deviation of the head and the eyes ipsilaterally to the lesion, ipsilateral orientation to the lesion when stimulated by the front or the side of the lesion and ignoring objects or people on the contrary side of the lesion ^11^. Anosognosia was characterized through questions about the motor deficit of the patient and confirmed only when no knowledge of the motor weakness was expressed, even after the demonstration made by the examiner ^17^. Severity of CVA was assessed through the NIH scale (National Institute of Health Stroke Scale - NIHSS)^18^.

For the diagnosis and graduation of Pusher syndrome, we used the Scale for Contraversive Pushing - SCP. Based on the observations made by Davies, SCP assesses: 1) symmetry of spontaneous posture, both seated and in orthostatic position; 2) extension of upper and/ lower limbs with the contact surface, both seated and in orthostatic position; and 3) resistance to passive correction of posture, both seated and in orthostatic position 11, 19. Pusher syndrome was confirmed if the patients presented all criteria, reaching a score of at least 1 in each criterion.

The present study was approved by the research ethics committee of our institution (protocol n. 11723 / 2003).

### Assessment of Vestibular Function

#### Caloric Stimulation

The caloric test was conducted according to the stimulation techniques of Fitzgerald and Hallpike^20^. Each ear was irrigated alternatively with constant water flow at 30°C and 44°C, during 40 seconds. The order of stimulation was: right ear and left ear with hot stimulation; left ear and right ear with cold stimulation. We gave an interval between stimulations so that there would be no accumulated effect. The electro-occulography was carried out with NEUROGRAFF - Eletromedicina - VENG digital, model VECWIN. Head position was corrected after each stimulation and maintained at 60° extension with verticalized Frankfurt line, allowing that the horizontal semicircular canals remained in the vertical position. We asked questions to patients or asked them to make calculations during the test to prevent the cortical inhibition effect over the vestibular system. All patients remained with a mask over their eyes to prevent eye opening and consequently nystagmus inhibition during the test. It was not possible to male ocular fixation owing to the level of consciousness and collaboration of the patients. No anti-vertigo medication had been taken two days before the otoneurological exams.

The maximum speed of the slow component of the nystagmus was analyzed after the irrigation. Directional preponderance (PD) was determined based on Jongkees^21^ method, and it was considered abnormal if over 25%. The speed of the slow component was considered normal if within the values 3 and 51/sec.

#### Rotation stimulation

The stimulation with rotation chair was made using the equipment Ototest Alvar Electronic - IV 2 TR, connected to the vectoelectronystagmographic digital system Neurograff. To prevent visual stimulus, the exam was performed in a dark room and patients’ eyes were closed by a mask. The head was position at 30° flexion so that horizontal semicircular canals could remain close to the stimulus plane (horizontal position). The patients were submitted to sinusoidal oscillations with accelerations from 18º/sec^2^ to complete stoppage of the chair. Similarly to the caloric test, we asked questions to the patients during data acquisition. Analyzed parameters were: slow phase of nystagmus and nystagmus frequency. The speed of slow phase was considered abnormal if below 7°/s or above 30°/s. Abnormality of nystagmus frequency was estimated through calculation of directional preponderance.

## RESULTS

We studied 9 patients, 5 men and 4 women, with mean age of 71.8 ± 5.9 years. Table 1 describes the demographical and clinical characteristics of patients. The assessments of vestibular function were made after a median time of 10 days after CVA ranging from 5 to 548 days. Five patients (55.55) presented encephalic lesions of the right cerebral hemisphere, and three of them had heminegligence and two, anosognosia. At the moment of the assessment, the patients presented mean NIHSS of 18.33 (variation between 13 and 24).

**Table 1:** Clinical and demographic data of assessed patients.

Patient	etiology	lesion side	heminegligence	anosognosia	hemianopsia	sensitivity	NIH scale
1	AVCh	R	no	No	Yes	hypo L	13
2	AVCi	R	no	No	no	anesthesia L	13
3	AVCh	R	Yes	No	Yes	hypo L	14
4	AVCi	L	no	NT +	no	normal	22
5	AVCi	R	Yes	Yes	Yes	hypo L	17
6	AVCh	L	no	NT *	no	anesthesia R	21
7	AVCi	L	no	NT *	no	normal	21
8	AVCh	L	no	NT *	no	normal	24
9	AVCi	R	Yes	Yes	Yes	hypo L	20

Key: AVCi: Ischemic cerebral vascular accident; AVCh: Hemorrhagic cerebral vascular accident; NT: Not tested.

R: right; L: left.

+ Patient was confused, did not cooperate with the exam.

* Aphasic patients.

Three patients presented contralateral directional preponderance to the encephalic lesion in the caloric test. The other patients presented labyrinthic normal function in this test (Table 2). In the caloric test, we found four patients with directional preponderance and we did not evidence correlation with side of the lesion (Table 3).

**Table 2:** Results of caloric test.

Patient	Lesion side	Odq	OEq	ODf	OEf	Preponderance
1	R	29,6	21,1	30,5	32,4	9,10%
2	R	6,5	13,3	3,9	2,8	29,6% left
3	R	18,5	8	29,3	29,4	12,40%
4	L	27,7	38,8	42,7	39,4	9,80%
5	R	19,4	12,4	14,2	20,1	19,3%
6	L	36,9	12,3	13,6	43,4	51% right
7	L	16,6	15,6	29,2	27,4	3,10%
8	L	5,1	3,4	Not performed	Not performed	19,6%
9	R	4,5	3,3	12,2	2,9	45,3% left

Key: ODq: right ear - hot stimulation; OEq: left ear - cold stimulation;

ODf: right ear - cold stimulation; OEf: left ear - cold stimulation

**Table 3:** Results of rotation test.

Patient	Lesion side	Clockwise (R) Vel (º/s)	Anti-clockwise (L) Vel (º/s)	Preponderance (%)	Direction (R) F (nystagmus/s)	Anti-clockwise (L) F (nystagmus/s)	Preponderance (%)
1	R	17	30.9	29% left	5	7	17%
2	R	6	5.3	6.2%	4	6	20%
3	R	54.8	21.8	43% right	6	5	9.09%
4	L	25.4	27	3%	AI	AI	———————
5	R	13.5	7.4	28.8% right	9	5	28,5% right
6	L	12.3	5.8	35.6% right	18	7	44% right
7	L	AI, large amplitude	AI, large amplitude	——————-	AI	AI	———————
8	L	Arreflexia	Arreflexia	——————-	Arreflexia	Arreflexia	———————
9	R	AI, dysmetria, decomposition of slow component	AI, dysmetria, decomposition of slow component	——————-	AI	AI	———————

Key: Direction (R): rotation to the right - clockwise direction; Anti-clockwise (L): rotation to the left - anti-clockwise direction;

Vel: speed of the slow component; F: nystagmus frequency; AI: impossible analysis.

## DISCUSSION

Up to recently, the influence of the vestibular system in the manifestations of Pusher syndrome had only been investigated by tests that assessed the otolithic function ^16^. In this aspect, the study of SVV demonstrates inclination to one side in patients with peripheral and central vestibular dysfunctions, a fact that does not occur in patients with Pusher syndrome ^16^. The results of the present study indicate that the dysfunction of the semicircular canals also seems to be essential for the expression of Pusher syndrome.

The caloric test is the best way to diagnose loss of unilateral vestibular function ^22^ and when added to the rotation test, it may also support the understanding of balance disorders. The confirmation of PD in a caloric test occurs when we observe significant asymmetry between duration of nystagmus to the right (caused by hot and cold stimulation to the left ear) and duration of nystagmus to the left (caused by hot stimulation to the left and cold to the right)^20^. PD can also be observed in the rotation test through the difference in angular speed of slow component or the difference in nystagmus frequency. The fact that PD of horizontal caloric nystagmus may occur without spontaneous nystagmus or significant nasal paresis indicates that PD is not produced only by disorders of peripheral vestibular system 22, 23. Prencht (1974) said that damage to the central nervous system (CNS) may cause PD through increase in dynamic sensitivity of neurons in the medial vestibular nucleus (NVM), and the consequent difference of nystagmus response ^24^. However, in the present study, we found a random pattern of caloric and rotation test affections among the patients with Pusher syndrome. These results suggest that horizontal semicircular canals do not participate in balance disorders of this condition.

PD values obtained in the caloric test were higher than in the rotation test. This difference may be resultant from the characteristics of the stimuli, given that in the caloric irrigation there is unilateral vestibular stimulation and in the rotation test, there is bilateral stimulation. However, the activity of NVM type IA neurons is directly modulated only on one side during the caloric stimulation, which is an activity directly modulated by both sides during the rotation stimulus ^22^.

The patients with directional preponderance in the caloric test, in most of the cases coincide with those that had PD in the rotation test (Tables 2 and 3). This characteristic was also observed in the study by Halmagyi et al. ^22^.

Studies by Mittelstaedt (1992) suggest the existence of a second graviceptive system that is based on afferences resultant from the renal capsule, large vessels and abdominal viscera, which would reach the central nervous system via phrenic or vagus nerve ^6^.

The findings of the present study favored the concept of a second graviceptive system, with interceptors afference and encephalic projections, which participate in the posture control found in patients with Pusher syndrome. The main foundation to this conclusion resides in the fact that no abnormalities of function of semicircular canals and otolytes were found in this condition.
